# Prevention of Brain Metastases: A New Frontier

**DOI:** 10.3390/cancers16112134

**Published:** 2024-06-04

**Authors:** Alessia Pellerino, Tara Marie Davidson, Shreyas S. Bellur, Manmeet S. Ahluwalia, Hussein Tawbi, Roberta Rudà, Riccardo Soffietti

**Affiliations:** 1 Division of Neuro-Oncology, Department of Neuroscience ‘Rita Levi Montalcini’, University and City of Health and Science Hospital, 10126 Turin, Italy; roberta.ruda@unito.it; 2 Department of Melanoma Medical Oncology, UT MD Anderson Cancer Center, Houston, TX 77030, USA; tmdavidson@mdanderson.org (T.M.D.); htawbi@mdanderson.org (H.T.); 3 Department of Medical Oncology, Miami Cancer Institute, Miami, FL 33176, USA; shreyas.bellur@baptisthealth.net (S.S.B.); manmeeta@baptisthealth.net (M.S.A.); 4 Candiolo Cancer Institute, FPO-IRCCS, 10060 Candiolo, Italy

**Keywords:** brain metastases, breast cancer, lung cancer, melanoma, prevention, prophylactic cranial irradiation, screening, secondary chemoprevention, targeted agents

## Abstract

**Simple Summary:**

This review discusses the topic of prevention of brain metastases from the most frequent solid tumor types, i.e., lung cancer, breast cancer and melanoma. Within each tumor type, the issues of screening in asymptomatic patients, prophylactic strategies with radiation and secondary chemoprevention with targeted agents are discussed.

**Abstract:**

This review discusses the topic of prevention of brain metastases from the most frequent solid tumor types, i.e., lung cancer, breast cancer and melanoma. Within each tumor type, the risk of brain metastasis is related to disease status and molecular subtype (i.e., EGFR-mutant non-small cell lung cancer, HER2-positive and triple-negative breast cancer, BRAF and NRAF-mutant melanoma). Prophylactic cranial irradiation is the standard of care in patients in small cell lung cancer responsive to chemotherapy but at the price of late neurocognitive decline. More recently, several molecular agents with the capability to target molecular alterations driving tumor growth have proven as effective in the prevention of secondary relapse into the brain in clinical trials. This is the case for EGFR-mutant or ALK-rearranged non-small cell lung cancer inhibitors, tucatinib and trastuzumab–deruxtecan for HER2-positive breast cancer and BRAF inhibitors for melanoma. The need for screening with an MRI in asymptomatic patients at risk of brain metastases is emphasized.

## 1. Introduction

Although early diagnosis and treatment has improved the prognosis in the last few decades, patients with brain metastases continue to have a short survival, even in cases of limited or stable extracranial disease [1]. One of the main reasons is that the brain is a sanctuary site for metastases due to the poor penetration through the normal blood–brain barrier (BBB) of most cytotoxic and targeted compounds, which leads to difficulty in targeting micrometastases [2]. Notably, BBB is normal in micrometastases (<1 mm), while the brain–tumor barrier (BTB) is leakier, as it lacks tight junctions and astrocyte–endothelial contacts. Prevention strategies were initially developed for tumors with a high propensity to relapse into the brain, such as small cell lung cancer (SCLC), and consisted of the so-called prophylactic cranial irradiation (PCI). This modality has been proven effective in reducing the risk of brain metastasis (BM) but at the price of cognitive decline in long-surviving patients. More recently, it has emerged that some molecular subgroups of patients with non-small cell lung cancer (NSCLC), breast cancer and melanoma have a higher propensity to develop brain metastases and, at the same time, are treatable with new effective inhibitors with an increased capacity to cross the BBB. Thus, preventive strategies using molecular agents (“chemoprevention”) are gaining increasing interest.

This article will review the old and novel approaches for the prevention of BM in lung cancer, breast cancer and melanoma, which are among the solid tumors that are major sources of BM.

A literature search was conducted of studies or reviews published in the English language from 1995 to 2023 in databases, such as Medline, Embase, Scopus and Web of Science. The list of ongoing clinical trials was derived from clinicaltrials.gov (accessed on 15 May 2024).

## 2. Brain Metastases from Lung Cancer

### 2.1. Risk in Relation to Disease Status and Molecular Subtype (Table 1)

Lung cancer is the leading cause of cancer-related mortality and frequently results in BM [3]. The incidence of BM shows a rising trend due to the advent and usage of better diagnostic modalities and therapy [4].

**Table 1:** Incidence of brain metastases in NSCLC.

Whole Population	15–20%
EGFR mutant	15–20%
KRAS G12C mutant	10–12%
ALK rearranged	4–5%
MET mutant	2–3%
BRAF V600E mutant	1–5%
HER2 exon 20 mutant	1–3%
RET mutant	1–2%
ROS1 mutant	1–2%
NTRK mutant	<1%

Approximately 20% of patients with stage IV (metastatic) or III (advanced) non-small cell lung cancer (NSCLC) have BM at initial diagnosis, and more than one-third develop BM as their disease progresses [5,6,7,8,9].

Age < 60 years and adenocarcinoma histology are known risk factors for developing BM.

The risk of developing BM varies according to the molecular subtype [10].

EGFR is a transmembrane glycoprotein with an extracellular epidermal growth factor binding domain and an intracellular tyrosine kinase domain that regulates signaling pathways to control cellular proliferation. The majority of genetic alterations occur as exon 19 deletions (60%) or L858R missense substitutions (35%), both of which result in constitutive activation of the receptor leading to cell growth and proliferation.

EGFR mutations comprise 10–15% and 25–50% of NSCLC in White and Hispanic or Asian populations, respectively [11,12]. The incidence of BM in patients with EGFR-mutant NSCLC has been reported in 29–30% of patients [10].

ALK is a gene whose rearrangement (most commonly translocations) induces the autophosphorylation and constitutive activity of ALK and downstream signaling cascades such as PI3K and RAS. In particular, RAS activation acts as an oncogenic driver through the dysregulation of the cell cycle, growth and metastases. ALK-translocated NSCLC represents 3–7% of all NSCLC patients and between 27% and 40% have BM at diagnosis [13].

KRAS mutations occur in 35% of all NSCLC patients and 31–39% develop BM [14]. ROS1 mutations account for 1% to 2% of NSCLC patients with 29% developing BM [10]. RET mutations occur in approximately 1–2% of NSCLC patients with 32% developing BM [15]. MET mutations are present in 2–4% of patients diagnosed with NSCLC [12,16], but there is lack of information on the risk of developing BM.

Nearly half of patients with small cell lung cancer (SCLC) develop BM [17,18]; however, molecular subgroups with a different propensity for the brain have not been identified [19].

### 2.2. The Issue of Screening

According to the NCCN guidelines (2024) [20] brain magnetic resonance imaging (MRI) with contrast is recommended at diagnosis to rule out asymptomatic BM in patients with stage II, III and IV NSCLC if aggressive combined modality therapy is being considered. Patients with stage I tumors are less likely to develop BM; therefore, brain MRI is optimal and can be considered for select patients of high risk (e.g., tumors > 5 cm, central locations). If a brain MRI cannot be performed, computer tomography (CT) of the head with contrast is an option. The EANO-ESMO guidelines (2017) make similar recommendations [21]. One must be aware that MRI has a superior sensitivity over CT, even when combined with positron emission tomography (PET) staging [22]. Conversely, brain MRI is not routinely recommended for routine surveillance in patients without symptoms regardless of tumor stage at diagnosis.

As for SCLC, the NCCN guidelines (2024) [20] recommend brain MRI (preferred) or CT with contrast in all patients. Brain MRI or CT with contrast are suggested for surveillance every 3–4 months during Year 1, then every 6 months afterwards regardless of PCI status.

### 2.3. Prophylactic Cranial Irradiation in SCLC

Prophylactic cranial irradiation has shown success in SCLC for more than 30 years. In a retrospective analysis on 54 patients receiving PCI, 96% were less likely to develop BM with a relative risk (RR) of 0.04 [23]. Overall, other studies showed that PCI achieves an RR between 0.18–0.73 in developing new BM [24,25,26,27,28,29,30,31,32,33,34,35].

A phase III trial run by the European Organization for Research and Treatment of Cancer (EORTC) group assessed the efficacy of PCI in patients with an extensive SCLC responsive to chemotherapy [36]. The PCI group (N = 143) reported an increased disease-free survival (RR of recurrence or death, 0.75, 95% CI, 0.65–0.86, *p* < 0.001) and decreased incidence of BM (RR, 0.46; 95% CI, 0.38–0.57, *p* < 0.001). The cumulative risks of symptomatic brain metastases at 6 and 12 months were 4.4% and 14.6%, in the irradiation group and 32.0% and 40.4% in the control group with an HR of 0.27 (0.16–0.44). At 6 months, the survival rate without disease progression was 23.4% (95% CI, 16.6–30.9) in the irradiation group and 15.5% (95% CI, 10.1–22.0) in the control group.

Yin et al. (2019) [37] showed in their meta-analysis of seven randomized trials that PCI significantly reduced the incidence of brain metastases compared to observation, reporting a hazard ratio (HR) of 0.45 (*p* < 0.001). The PCI group also reported a prolonged overall survival (OS) with an HR of 0.81 (*p*  <  0.001). Another meta-analysis of 63 studies [38] corroborated this finding in 8906 patients with SCLC who received PCI, reporting a 55% risk-reduction in developing new BM with a relative risk (RR) of 0.45 (*p* < 0.001). Of those, 5470 patients with limited SCLC reported an RR of 0.45 (*p* < 0.0001) and 1763 patients with extensive disease reported an RR of 0.51 (*p* = 0.01). A lower incidence of BM was reported in 912 patients who achieved a complete response at restaging, confirmed by magnetic resonance imaging (MRI) with an RR of 0.51 (*p* = 0.047). However, this meta-analysis suggests that PCI might infer a therapeutic rather than a preventative benefit, as it was shown that PCI confers a survival advantage only when considering radiologically confirmed BM with an HR of 0.59 (*p* < 0.001). Conversely, in patients with an MRI-confirmed absence of BM at restaging, survival did not significantly differ in patients who received PCI vs. those who did not—demonstrating an HR of 0.74 (*p* = 0.08).

PCI also increases the risk of late neurocognitive decline. Sixty-two patients enrolled in the RTOG0212 trial who received PCI at 18 Gy and 85–89% who received PCI at 36 Gy developed neurocognitive decline in at least one neurocognitive test [39]. Therefore, to mitigate neurocognitive decline, WBRT with hippocampal sparing (HS) is being studied. The ongoing NRG Oncology CC003 is a phase II/III trial aimed at assessing the efficacy of WBRT with HS compared to WBRT in patients with SCLC [40].

### 2.4. Prophylactic Cranial Irradiation in NSCLC 

The phase III NVALT-11/DLCRG-02 trial showed that PCI reduces the incidence of symptomatic brain metastases. Patients were randomized to the PCI group (n = 86) and observation group (n = 88). In total, 6 of 86 patients (7%) in the PCI group developed symptomatic BM vs. 24 of 88 patients (27.2%) in the control group (*p* < 0.001) at 2 years. PCI also significantly increased the time to develop symptomatic BM with an HR of 0.23 (*p* = 0.0012), although no survival benefit was seen. The median OS was marginal in the PCI arm: 24.2 months vs. 21.9 months, respectively (*p* = 0.56) [41].

The phase III NRG Oncology/RTOG 0214 trial studied PCI in surgically resected or pretreated locally advanced NSCLC [42]. The final analysis included 340 patients with a median follow-up of 2.1 years. The study corroborated the results of the NVALT-11/DLCRG-02 trial, showing a reduction in the cumulative incidence of BM with no survival benefit. The DFS at 5 years and 10 years was 19.0% and 12.6% for PCI and 16.1% and 7.5% for observation, respectively, with an HR of 0.76 (*p*  =  0.03). However, a subgroup analysis reported an OS benefit in those patients who received PCI but did not receive surgery: the median survival time was found to be 2.3 years compared to 1.9 years (*p* = 0.03). The incidence of BM at 5 and 10 years was 16.7% vs. 28.3% for PCI vs. observation with an HR of 0.43 (*p* = 0.004). The PCI group was 57% less likely to develop BM. Interestingly, in the no-surgery cohort, younger patients (<60 years) and those with squamous histology reported a higher incidence of BM. A meta-analysis pooling seven studies also reported similar findings [43]. In 1462 patients, the cumulative incidence of BM was reduced by 58% after sensitivity analysis (HR 0.42). However, PCI did not impact the incidence in patients with a poor functional status (HR 0.51). Overall survival was also not significantly influenced by the administration of PCI (HR 1.01). Another meta-analysis [44] reported a 13% reduction in the incidence of BM in patients who received PCI. Patients in the PCI group were also one-third as likely to develop BM vs. patients in the observation group (RR, 0.33).

Therefore, PCI is not a guideline management for NSCLC, since it offers no survival benefit.

### 2.5. Secondary Chemoprevention

In recent years, the molecular profiling of NSCLC has resulted in the discovery of driver mutations and, subsequently, therapies targeting such mutations. Since there are limited data on the primary prevention of brain metastases, we have focused on secondary prevention (Table 2).

A lower CNS progression rate following first-generation gefitinib and erlotinib as first-line in brain metastases from EGFR-mutated NSCLC in comparison with chemotherapy (33% vs. 48%) has been reported [45]. Osimertinib is a potent third-generation EGFR TKI, which also inhibits the EGFR T790M resistant mutation. The phase III FLAURA trial assessed osimertinib vs. standard EGFR TKI in NSCLC and reported 22% of patients with new brain lesions on osimertinib vs. 41% on standard EGFR TKI, and 5% of patients progressed to develop BM on osimertinib vs. 12% in the control arm [46].

Crizotinib is a small-molecule TKI of ALK, ROS1 and MET kinases [47]. In the phase III PROFILE1014 study, which compared crizotinib with standard platinum-based chemotherapy in patients with ALK-NSCLC, crizotinib achieved a significantly better rate of intracranial disease control compared with cytotoxic chemotherapy. However, there was no significant difference in the time to CNS progression between the two treatments in 263 patients without baseline BM [48]. This may be partly attributed to the poor CNS penetration of crizotinib and p-glycoprotein-mediated efflux through the blood–brain barrier (BBB) [49]. Alectinib is a highly selective ALK inhibitor that has shown excellent CNS penetrance compared to crizotinib in a phase III trial on ALK-rearranged NSCLC [50]. The time to CNS progression was significantly longer in the alectinib group compared to the crizotinib group with a cause-specific HR of 0.16 (*p* < 0.001) and 12% progressing to a CNS event with alectinib vs. 45% with crizotinib. The cumulative incidence of CNS progression was significantly lower with alectinib, with a 12-month rate of 9.4% vs. 41.4% with crizotinib. The HR for death or disease progression in patients with baseline BM was 0.40, and in patients without baseline BM was 0.51, favoring alectinib for therapy. Brigatinib is a next-generation ALK inhibitor with activity in crizotinib-resistant NSCLC. In the ATLA-1 L trial, 9% of patients in the brigatinib group and 19% in the crizotinib group had intracranial disease progression as the first site of disease progression. In patients without brain metastases at baseline, 1% in the brigatinib group and 5% in the crizotinib group had intracranial disease progression as the first site of disease progression. The HR for death or disease progression was 0.27, favoring the use of brigatinib [51]. The single-arm phase II ASCEND 7 trial studied ceritinib in patients with active BM based on prior exposure to ALK inhibitors (ALKi) and radiation. The location of first disease progression was reported to be intracranial in 31% in Arm 1 (prior RT + ALK I), 60% in Arm 2 (no priori RT+ prior ALK I), 16.7% in Arm 3 (prior RT + no prior ALK I) and 50% in Arm 4 (no prior RT or ALK I) [52]. The CROWN trial assessed the efficacy of lorlatinib, an oral ALK inhibitor with excellent CNS penetrance, and reported that the time to CNS progression was significantly longer compared to crizotinib. In total, 96% of patients were alive without CNS progression at 12 months in the lorlatinib group vs. 60% in the crizotinib group with an HR for intracranial progression of 0.07. The cumulative incidence of CNS progression as the first event was 3% with lorlatinib and 33% with crizotinib (HR, 0.06) [53].

CodeBreaK200 assessed sotorasib as a KRAS inhibitor in comparison to docetaxel in patients with pretreated NSCLC BM. The study showed that the median time to BM recurrence in patients with previous CNS disease was prolonged in the sotorasib group compared to the docetaxel group (15.8 months vs. 10.5 months) with an HR of 0.52, but without reaching statistical significance [54]. The KRYSTAL-1 single-arm trial assessed adagrasib, an irreversible inhibitor of KRASG12.C, with increased CNS penetrance [55]. The median intracranial PFS in the 42 patients with BM at baseline was 5.4 months [56]. However, there were no data available on the time to CNS metastases.

A pooled analysis of the ALKA-372-001, STARTRK-1 and STARTRK-2 trials assessed entrectinib in patients with ROS1-driven NSCLC BM. The time to CNS progression was 13.6 months in patients with investigator-assessed baseline CNS metastases. New lesions were reported in 4.5% of patients with absent BM at baseline. The CNS progression risk was reported to be 39% in patients receiving entrectinib at 12 months [57].

A pooled analysis of the LIBRETTO-001 trial or the LIBRETTO-201 expanded access program was performed [58]. Patients with absent baseline BM exhibited no CNS progression for the duration of therapy. However, 12 patients experienced only extracranial progression and 23 patients with baseline BM experienced progression, with 3 patients experiencing CNS-only progression. The cumulative incidence rates for CNS progression at 6, 12, 18, 24 and 36 months were 3%, 10%, 17%, 17% and 20, respectively [58]. Patients with CNS disease at baseline were significantly more likely to progress in the CNS than those without baseline CNS disease (*p* = 0.01).

**Table 2:** Post-hoc analysis of clinical trials on targeted agents in NSCLC: CNS relapse after first-line treatment.

Treatment Arm	Mutation Targeted	Brain Metastases	Time to CNS Relapse	Other Parameters of Secondary Prevention
Osimertinib [46]	EFGR	Absent or stable	N/A	Patients with new BM: 5%
Crizotinib [49]	ALK	Absent or pretreated + stable	N/A	Median time to intracranial progression: NR in both HR 0.69
Alectinib [50]	ALK	Absent or stable	HR: 0.16 12-month rate: 9.4%	N/A
Brigatinib [51]	ALK	Absent or treatment-naïve + stable	N/A	CNS as first site of progression: 9% No BM at baseline, 1%
Ceritinib [52]	ALK	Active	N/A	CNS as first site of progression: Arm 1 (prior RT + ALKi): 31% Arm 2 (no prior RT + prior ALKi): 60% Arm 3 (prior RT + no prior ALKi): 16.7% Arm 4 (no prior RT or ALKi): 50%
Lorlatinib [53]	ALK	Stable + treatment naïve or active + pretreated	N/A	No CNS progession at 12 months: 96% HR for intracranial progression of 0.07 CNS as first site of progression: 3% HR 0.06
Sotorasib [54]	KRASpG12C	Absent or stable	15.8 months HR of 0.52	N/A
Entrectinib [56]	ROS1	Stable	13.6 months	New CNS lesions: 4.5% (absent BM at baseline) CNS progression risk at 12 months: 39%
Selpercatinib [58]	RET	Stable or active with 14 days of stable symptoms, scans and steroid dosage	N/A	No BM at baseline: No CNS progression BM at baseline: 10% Cumulative incidence rates for CNS progression 6-months: 3% 12 months: 10% 18 months: 17% 24 months; 17% 36 months: 20%

CNS: central nervous system; ALKi: ALK inhibitor; RT: radiotherapy; N/A: not applicable.

Capmatinib, a selective MET inhibitor, was investigated in the GEOMETRY trial. However, the number of patients accrued with BM was very limited, as well as the progression data. Out of 13 patients with evaluable BM, 92.3% achieved disease control [59]. There was no subset analysis looking at CNS progression.

Ongoing clinical trials on BM from NSCLC are reported in Table 3.

## 3. Brain Metastases from Breast Cancer 

### 3.1. Risk in Relation to Disease Stage and Molecular Subtype (Table 4)

HER2 is a membrane tyrosine kinase that is part of the EGFR family. HER2 expression is upregulated in brain metastases compared to primary tumors and is involved in the colonization of the brain by breast cancer cells. HER2 upregulation promotes cellular survival and proliferation through multiple downstream pathways.

**Table 4:** Incidence of brain metastases in breast cancer.

Full population (metastatic BC)	10–25%
HER2+	20–49%
Triple negative	15–39%
ER+ HER2+	34%
ER+ HER2−	19%

The risk of brain metastasis (BM) in early or locally advanced (non-metastatic) BC is low. Overall, patients with BM as the site of first recurrence represent 0.7–5.5% with an incidence per year of median follow-up of 0.1–3.2%, with the highest frequency in women with inflammatory disease [60]. Regarding Grade 1 or 2 ER+ HER2− patients, no cases of BM as the first site of metastatic disease have been reported [61,62,63]. Regarding HER2-positive patients, those with BM as the site of first recurrence represent 0.8–12.2% with an incidence per median year of follow-up ranging from 0.2% to 3% [64,65,66]. Regarding triple-negative patients, those with BM as the site of first recurrence range between 1.7% and 4.7% with an incidence per year of median follow-up of 0.4–1.6% [67,68,69,70].

Conversely, the risk of BM in metastatic BC (MBC) is definitely higher as compared to non-metastatic disease. In most studies the prevalence predominantly refers to symptomatic BM; however, a number of asymptomatic patients have been found in the context of restaging. Overall, the values range from 10% to 25% [71,72,73,74]. In the ESME cohort (a real-life cohort describing the daily practice in French cancer centers and hospitals), the prevalence is significantly higher in HER2-positive and triple-negative patients (49% and 38%, respectively) as compared to ER+ HER2− (19%) and ER+ HER2+ (34%) [75]. Importantly, the development of BM in triple-negative BC patients commonly occurs with concurrent extracranial progression, while in HER2-positive patients it occurs in the setting of a stable systemic disease (due to the efficacy of anti-HER2 strategies). In other series, patients with HER2+ disease developing BM range from 20% to 42% with a cumulative incidence at 1 or 2 years of 10–31% [67,72,76,77,78,79,80,81,82,83,84].

Patients with triple-negative disease developing BM range between 15% and 37% with a cumulative incidence at 1 and 2 years of 11–19% [72,85,86].

### 3.2. The Issue of Screening

There is a paucity of studies investigating the usefulness of screening procedures to detect asymptomatic BM in MBC. The studies are largely retrospective in nature, often of small size, employing both CT and MRI and with a variable follow-up duration [80,81,87]. The detection rate varied between 6% and 34% and confirmed the higher risk for patients with HER2+ and triple-negative BC patients. The lack of well-designed prospective studies explains why an MRI screening for asymptomatic BM in metastatic BC patients is not routinely recommended in the ESMO and NCCN international guidelines [88,89]. However, they suggest to devote particular attention to HER2+ and triple-negative patients, especially if the detection of CNS metastases will alter the choice of systemic therapy.

In general, there are pros and cons regarding the usefulness of screening strategies. Identifying and treating asymptomatic BM might prevent neurologic complications, thus improving QoL and reducing the need for WBRT or surgery. Recent studies have reported a modest advantage in survival for patients with asymptomatic BM [90,91,92,93,94]. However, an old study on a small number of patients did not show a difference in survival between patients with asymptomatic versus symptomatic brain metastases [95].

Conversely, screening may carry an inherent risk of “over-diagnosis”, the exacerbation of patients’ anxiety and adverse effects of an early treatment with stereotactic radiosurgery. Moreover, the economic costs of frequent screening MRI studies and early stereotactic treatments are unknown.

Four studies are investigating the value of a systematic radiological screening in patients with either HER2-positive or triple-negative patients. Two trials (NCT03881605, NCT04030507) are randomizing patients to receive either MRI or clinical surveillance for BM every 4 months for 1 year. A single-arm observational trial (NCT03617341) is exploring the value of MRI at the time of initial diagnosis, first- and second-line treatment failure. The fourth study is a randomized trial (NCT00398437) comparing a brain MRI every 4 months versus once every 12 months.

### 3.3. Secondary Chemoprevention 

#### 3.3.1. Post-Hoc Analysis of Clinical Trials Aimed to Evaluate the Efficacy of Targeted Agents in Patients with Active or Stable Brain Metastases (Table 5)

All available data come from trials on HER2-positive MBC with BM receiving small tyrosine kinase inhibitors (TKIs), monoclonal antibodies (MoAs) and antibody–drug conjugates (ADCs).

Lapatinib is a first-generation TKI that inhibits both HER2 and EGFR. In the single-arm phase II study LANDSCAPE on untreated BM, the median time to CNS progression was 6 months [96]. The phase III CEREBEL trial was designed to address the issue of the best CNS protective effect between lapatinib–capecitabine and trastuzumab–capecitabine in a population without BM. No difference was seen regarding the primary endpoint with 3% of a CNS first site of relapse in the lapatinib–capecitabine arm compared with 5% in the trastuzumab–capecitabine arm [97]. Neratinib is a second-generation irreversible pan-HER TKI. In the randomized phase III trial NEfERT-T, the neratinib–paclitaxel combination was compared with the trastuzumab–paclitaxel combination in a first-line metastatic setting [98]: the incidence of CNS relapse in the neratinib arm was lower (HR 0.48) and time to CNS metastases delayed (HR 0.45). The phase III NALA trial randomized patients who had received two lines or more of HER-2-directed therapies between neratinib–capecitabine and lapatinib–capecitabine [99]: the incidence of intervention for symptomatic brain metastases decreased from 29% in the lapatinib–capecitabine arm to 23% in the neratinib–capecitabine arm. Overall, these data are consistent with a higher, even if modest, preventive effect of neratinib in comparison to lapatinib. Tucatinib is a selective HER-2 inhibitor, with a good penetration of the BBB and distribution into the CSF [100]. This drug, in combination with trastuzumab and capecitabine, has shown in the randomized phase III HER2CLIMB have an impressive efficacy in patients with MBC who had previously received trastuzumab, pertuzumab and T-DM1. In particular, 25% of patients with BM at inclusion (n = 291), both active and stable, did not progress at 1 year after tucatinib combination treatment versus 0% after placebo combination (*p* < 0.001), with a 68% reduction in the risk of CNS-PFS in the tucatinib arm [101]. In an updated exploratory analysis [102], the risk of developing new brain lesions as the site of first progression or death was reduced by 45.1% in the tucatinib combination group (HR 0.55). Pyrotinib is an irreversible pan-HER receptor inhibitor targeting HER1, HER2 and HER4. Pyrotinib + capecitabine has shown superiority over lapatinib + capecitabine in the phase III PHOEBE trial in MBC [103]; and in a recent single-arm phase II study on patients with HER2-positive MBC and BM [104], a PFS of 11.3 months for patients who were radiotherapy-naïve and 5.6 months for those progressive after radiotherapy was reported.

Pertuzumab is a monoclonal antibody that inhibits the dimerization of HER2. In the phase III CLEOPATRA trial, patients with HER2-positive MBC were randomized to receive in the first line either pertuzumab in combination with trastuzumab and docetaxel or placebo-trastuzumab–docetaxel. All patients were free of brain metastasis at inclusion. Overall, 13% of patients in both arms developed BM as the site of first relapse [105]. Interestingly, in patients receiving pertuzumab the relapse in the brain was delayed (15 months) as compared with patients with placebo (12 months), and the overall survival was superior (34 months versus 24 months) even if not statistically significant.

**Table 5:** Post-hoc analysis of clinical trials on targeted agents in breast cancer: CNS relapse after first-line treatment.

Treatment	Molecular Subgroup	Brain Metastases	CNS Relapse	Time to CNS Relapse
Lapatinib + Capecitabine [96]	HER2+ MBC	Untreated	NR	6 months
Lapatinib−Capecitabine vs. Trastuzumab−Capecitabine [97]	HER2+ MBC	Absent	3% 5%	5.7 months 4.4 months
Neratinib−Paclitaxel vs. Trastuzumab−Paclitaxel [98]	HER2+ MBC	Absent or Stable	8.3% 17.3%	NR NR
Neratinib−Capecitabine vs. Lapatinib−Capecitabine [99]	HER2+ MBC	Absent or Stable	23% 29%	NR NR
Tucatinib combination vs. Placebo combination [101]	HER2+ MBC	Active or Stable	NR, NR	13.8 months vs. 24.9 months
Pertuzumab combination vs. Placebo combination [105]	HER2+ MBC	Absent	13% 13%	15 months 12 months
Trastuzumab emtansine vs. Lapatinib−Capecitabine [106]	HER2+ MBC	Absent	2% 1%	NR NR

T-DM1 consists of trastuzumab conjugated with the microtubule inhibitory agent emtansine (DM1).

Trastuzumab emtansine is an ADC that was compared to a lapatinib and capecitabine combination in the phase III EMILIA trial as a second-line treatment of HER2-positive MBC after failure of trastuzumab and pertuzumab. In an exploratory post-hoc analysis on patients without brain metastases at inclusion, CNS relapse occurred in 2% of patients receiving T-DM1 as compared to 1% in those receiving a lapatinib–capecitabine combination (not statistically significant) [106].

Trastuzumab deruxtecan (Tdx-D) is a novel ADC consisting of trastuzumab conjugated with the topoisomerase1 inhibitor deruxtecan, which has shown a high efficacy against brain metastases in some non-randomized trials [107,108]. However, no data are available thus far on a preventive effect of this drug.

#### 3.3.2. Prospective Studies

There are few ongoing prospective studies whose design consists of a selection of patients with a limited number of BMs successfully treated with surgery or radiotherapy to receive an agent with the aim of targeting the micrometastatic disease. An example is the US phase I/II randomized trial NCT03190967 in previously locally treated BM from HER2-positive MBC, which is comparing T-DM1 or Tdx-D alone to T-DM1 or Tdx-D plus temozolomide (TMZ) [109]. The “preventive” drug of the combination is temozolomide, an alkylating agent with a good penetration of the normal BBB due to its lipophilicity and low molecular weight, which is registered for the treatment of glioblastoma and recurrent anaplastic astrocytoma. This compound is not active in metastatic breast cancer [110], but in murine models of breast cancer demonstrated the capability to significantly prevent the outgrowth of BM and extend survival [111]. Conversely, consistent with the clinical data, no effect was seen in established brain metastases in the preclinical model. Prevention was achieved by using TMZ in a metronomic schedule instead of the traditional regimen used in glioblastomas with high doses for a limited duration. In the aforementioned clinical trial design, T-DM1 or Tdx-D, both active agents in HER2-positive brain metastases from MBC [107,112], were added to TMZ to evaluate the activity of the combination in a cohort of HER2-positive patients with BM from MBC who have been treated with surgery and/or SRS and/or WBRT. The primary endpoint is freedom from distant brain metastases at 1 year (increase from 50% to 65%), measured by the rate of brain relapse-free survival (RFS). Secondary endpoints are those typical of trials on BM (intracranial and systemic PFS and OS). The preliminary results of the phase I part have been recently published [113]. Twelve patients were enrolled in June 2021 and are evaluable. The phase I part has established the optimal dose for TMZ to be used and, overall, the safety of the treatment combination was acceptable. In total, 10 patients without new distant brain lesions left the study (3 patients for local or leptomeningeal progression, the others for toxicity), while only 2 patients (16%) presented with new BM (1 at 17.8 months and 1 at 1.8 months from inclusion into the study). Moreover, the authors extracted cfDNA from plasma and CSF and analyzed the mutational profile by whole-exome sequencing. Two findings are noteworthy. First, 6 out of 12 patients had cancer-linked DNA mutations in the CSF despite a brain MRI not showing active lesions at study inclusion, thus suggesting an ongoing brain metastatic colonization. Second, in 14% of CSF samples, the mutations were different from those in the matched plasma, suggesting clonal evolution in the CNS.

Another ongoing phase II randomized study (NCT05689619) is ongoing in Italy, comparing silibinin (an oral STAT3 inhibitor) with a placebo in single brain metastasis from breast cancer and NSCLC following complete resection. The primary endpoint is the time to intracranial local recurrence, with secondary endpoints such as intracranial distant recurrence, intracranial and systemic PFS, OS and QoL. The exploratory objectives will look at STAT3 downstream pathways in blood and/or CSF. The rationale of the study is represented by the role of STAT3 activation in reactive astrocytes in brain metastasis in both preclinical and clinical models [114,115], which represents a negative prognostic factor. STAT3 inhibition by the nutrient silibinin has been shown to be able to suppress breast cancer cell growth in preclinical models [116] and induce responses in patients with BM from NSCLC [117].

Ongoing clinical trials on HER2 + breast cancer BM are reported in Table 6.

## 4. Brain Metastases from Melanoma 

### 4.1. General Concepts

Skin cancers are the most commonly diagnosed group of cancers worldwide, and cutaneous melanoma accounts for approximately 1 in 5 of these malignancies [118]. Although not the most common, melanoma is by far the most lethal of the skin cancers and incidence rates are projected to increase globally over the next 20 years [118]. Impacting the lethality of the disease, melanoma has the third-highest incidence of brain metastasis among all types of cancer, following lung cancer and breast cancer [119].

Prior to 2011, there were limited available treatments for metastatic melanoma which made it almost universally fatal. Since that time, multiple new therapeutic classes including immunotherapies and targeted therapies have been approved which have changed the melanoma landscape and greatly improved outcomes for patients with both early and advanced stages of disease. Unfortunately, many of the landmark trials for these practice-changing therapeutics initially excluded patients with central nervous system (CNS) disease which has led to a delay in our understanding of treatment and prevention in melanoma brain metastasis (MBM).

Despite the initial exclusion of melanoma patients with CNS involvement from these landmark trials, we have seen an improved prognosis for patients with MBM since the addition of immunotherapy and targeted therapy. Patients with MBM diagnosed between the late 1980s to early 2000s had a median survival of 3–4 months [120,121,122,123,124]. Since 2011, patients diagnosed with MBM have an increased median survival of 5–6 months [125,126,127,128]. Data suggest an even greater improvement to 13 months in the last few years [129]. The Graded Prognostic Assessment (GPA) Index can be used to prognosticate individual patient outcomes with BM for multiple tumor types including melanoma [130]. The GPA index uses multiple prognostic factors to calculate an eligibility quotient (EQ) which predicts the probability of a patient’s survival for 12 months from the current date. The initial GPA index was initially designed using a cohort of patients diagnosed between 1985 and 2005. This index has since been revised to the current version (Melanoma-molGPA), with a larger population diagnosed between 2006 and 2015 [130]. The EQ is calculated based on the following factors which affect the overall survival of patients with MBM: age above or below 70, Karnofsky Performance Score (KPS), presence of extracranial metastasis, number of brain lesions and BRAF mutation status [131]. The Melanoma-molGPA is currently being used as a set of eligibility criteria in some clinical trials to identify patients likely to benefit from inclusion in a study despite CNS involvement.

### 4.2. Risk of Brain Metastases in Relation to Stage of the Disease and Primary Characteristics (Table 7)

There are limited historical data on the incidence of CNS involvement for many malignancies, including melanoma, due to a prior lack of mandated reporting of BM to federal agencies and population-based registries [132]. A recent study using Surveillance Epidemiology and End Results (SEER) data from 2016 on the presence of brain metastasis at the time of cancer diagnosis defined the following cumulative incidence rates as the number of patients diagnosed with brain metastases and a specific primary cancer divided by the total number of individuals diagnosed with that primary cancer [132]. Patients diagnosed with melanoma at any stage had a modest incidence portion of 0.65, but patients with metastatic disease only had a significant incidence portion of 28.16 [132]. Multiple additional studies consistently show that patients with stage IV melanoma are at a much higher risk for MBM than early-stage patients [128,129,133,134]. An autopsy study from 1978 of 216 patient with metastatic melanoma noted that 54% of patients had brain metastasis at the time of their death [135]. Additional studies concur that approximately 50% of patients with stage IV melanoma will develop BM [120,121,122,123,124,136,137,138].

**Table 7:** Incidence of brain metastases in melanoma.

Full Population	10%
**By Stage**	
I and II	5–10%
III	15%
IV	40–60%
**Other subtypes**	
Acral or Mucosal	10–30%
Uveal	2–6%
**Mutation Status**	
BRAF mutant	20–25%
KRAS mutant	20–25%

In terms of regionally advanced disease, patients with stage IIIC disease are more likely to develop MBM than stage IIIA or IIIB, with stage IIIC patients having about a 15% risk over their lifetime [127]. Additionally, two large databases of melanoma patients from the United States and Australia show the incidence of MBM diagnosis for stage III melanoma patients is 3.6% at 1 year, 9.6% at 2 years and 15.8% at 5 years from the time of initial melanoma treatment [139].

The diagnosis of MBM is not likely to not occur at the time of initial diagnosis [126,132] 90% of patients with MBM have extracranial metastases at the time of brain metastasis diagnosis with 40% of patients having greater than two extracranial sites [126]: overall 61% of patients are progressing in extracranial sites at the time of brain metastasis diagnosis. Only 10–15% of melanoma patients have brain metastasis as their first visceral disease site [140]. Thus, MBM is frequently associated with an increased disease burden and a more advanced stage of disease.

Other than stage, there has been direct contradiction as to whether other primary melanoma characteristics predict an increased risk of MBM development: head and neck or trunk primaries, ulceration status, lymph node macrometastases or in-transit lesions have been the most controversial, with inconsistent results [121,127,133,134,141].

Advanced-stage acral and mucosal melanoma subtypes have a lower risk for the development of brain metastasis (10–30%), although the data are significantly more limited than for cutaneous melanoma due to the rates of disease [134,142]. Uveal melanoma, which is genetically distinct from cutaneous melanoma, is primarily hematogenous in its metastatic spread, with 90% of metastasis to the liver [143]. Uveal cancer patients have a much lower risk (2–6%) of developing MBM [134,144].

In terms of patient characteristics, male patients are nearly 1.5 times more likely than female patients to develop MBM which has been supported by numerous studies [121,133,134,145]. The cause of this difference is unknown.

### 4.3. Risk of Brain Metastases in Relation to Molecular Subtypes and the Efficacy of Molecular Agents in Clinical Trials to Prevent Secondary Relapse into the Brain

BRAF is a protein kinase involved in the RAS-RAF-MEK-MAPK signaling pathway that regulates cell proliferation, differentiation, apoptosis and immunity. Somatic BRAF mutations occur in about 50% of melanoma patients [146]. Two studies have explored the effect of BRAF mutations on the risk of MBM development. Maxwell et al. (2017) [147] reported that BRAF-mutated patients had a significantly higher risk of developing MBM than BRAF wild-type patients (OR 2.24; 95% CI, 1.10–4.58). However, in a subgroup analysis of BRAF-mutant patients who had received any BRAF inhibitor treatment, the risk became equal to that of BRAF wild-type patients (OR 1.02; 95% CI, 0.40–2.60). Gardner et al. (2017) [133] noted a similar risk of developing brain metastases in both BRAF-mutant and wild-type patients; however, they did not provide information on which of those BRAF-mutated patients received treatment with BRAF inhibition. A combination of these studies suggests a potential MBM prevention benefit using BRAF inhibitor therapeutics for BRAF-mutant melanoma patients. More studies need to be conducted in this area. It should be noted that BRAF-positive patients diagnosed with MBM have a longer median survival than BRAF-negative patients (13 months vs. 9 months from the time of MBM diagnosis) [148].

The RAS-RAF-MEK-MAPK signaling pathway can also be activated by mutations in NRAS which are found in 15–25% of melanomas. Patients with either NRAS or BRAF mutations are more likely to have MBM at the time of diagnosis (*p* = 0.0076) [149]. An additional study of patients with MBM as their first site of visceral disease noted significant enrichment for KRAS mutations (8% pf patients) and noted that the primary lesions in these patients were thinner and without ulceration [150]. Overall, these data suggest that multiple mutually-exclusive mutations in RAS-RAF-MEK-MAPK increase the risk for MBM development.

### 4.4. Screening Data and Guidelines

Based on the National Comprehensive Cancer Network (NCCN) guidelines, brain imaging (MRI) at the time of diagnosis is not recommended for stage IA to stage IIIA melanoma, it should be considered for stage IIIB, IIIC and IIID melanoma and it is recommended for stage IV melanoma [NCCN Melanoma, 2024] [151]. While on active treatment, brain imaging remains a consideration with no consensus on the exact timing and without an increased frequency for any molecular subtype or for any particular primary melanoma characteristics including sites of disease or mutations detected [NCCN Melanoma, 2024] [151]. In terms of surveillance, NCCN guidelines recommend periodic brain MRI for up to 3 years in patients with stage IIIB or higher disease, but no further guidance is provided in terms of exact timing [NCCN Melanoma, 2024] [151].

The European Society for Medical Oncology (ESMO) guidelines, however, recommend brain MRI at the time of diagnosis for all patients with stage IIB melanoma and higher [152]. There is no ESMO consensus on the recommended timing of brain imaging while on active treatment or surveillance [152].

The lack of consensus globally on the timing for brain imaging in patients with melanoma on active treatment and surveillance leads to extreme variation in practice.

### 4.5. Ongoing Prospective Studies on Prevention (Table 8 and Table 9)

There are some very promising current preclinical developments in the treatment of MBM using the P13K/AKT/mTOR(PAM), STAT, s100A4 and the neurotrophin–heparanase pathway inhibitors; however, there is little work currently active in the area of MBM prevention [153,154,155]. This continues to be an unmet need.

Melanoma cells express high levels of neurotrophin receptors at the cell surface which, when activated by neurotrophins from within the intracranial compartment, cause the release of heparinase, allowing breakdown and access of the cancer cells into the BBB [153,154] Additionally, the STAT3 receptor is significantly increased in melanoma brain metastasis compared to melanoma cells from the primary site. STAT3 upregulates interleukin (IL)-23, which stimulates melanoma cells to secrete multiple signaling proteins key in parenchymal invasion and neoangiogenesis [153,154]. Studies have also suggested that the S100A4 protein is similarly involved in MBM neoangiogenesis [155].

The P13K/AKT/mTOR(PAM) pathway is upregulated in MBM when compared to primary tumors and other extracranial metastases [156]. This pathway commonly regulates cell proliferation and is felt to play a key role in the promotion of MBM through the upregulation of VEGF (critical for neoangiogenesis) and chemokine receptor type 4 (CCR4) which is a transmembrane receptor felt to be key in allowing melanoma cells to adhere to cerebral endothelium [154,157,158]. Preclinical data have shown decreased extravasation through the blood–brain barrier by melanoma cells and lower rates of brain metastasis development in mice treated with a low-dose preventative brain-penetrant dual PI3K/mTOR inhibitor [159]. Unfortunately, Buparlisib, a brain-penetrant PI3K inhibitor, showed no clinical intracranial response as a single agent in untreated MBM but could perhaps find some benefit in a preventative setting [160].

Despite the current lack of clinical trials focused on the prevention of MBM, it should be noted that as MBM is more common in later-stage disease, continued improvement in the neoadjuvant and adjuvant setting for early-stage melanoma, as well as better front-line treatment in advanced disease, will improve the prevention of MBM.

**Table 8:** Post-hoc analysis of clinical trials on targeted and immunotherapy agents in melanoma: duration of intracranial response in brain metastases.

Treatment	Molecular Subgroup	Brain Metastases	Intracranial Response Rate	Duration of Intracranial Response
Pembrolizumab [161]	None	Untreated	26%	Not reported
Nivolumab [162]	None	Untreated	20%	2.5 months
Ipilimumab + Nivolumab [163]	None	Untreated	54%	NR
Dabrafenib + Trametinib [164]	BRAF V600E mutant	Untreated	58%	6.5 months
Vemurafenib + Cobimetinib + Atezolizumab [165]	BRAF V600E mutant	Untreated	42%	7.4 months

**Table 9:** Ongoing trials on the systemic treatment of brain metastases from melanoma.

Study Name	Phase	NCT Number
BRAF targeted		
Encorafenib and Binimetinib Before Local Treatment in Patients with BRAF Mutant Melanoma Metastatic to the Brain	Phase II	NCT03898908
E6201 (MEK1 inhibitor) and Dabrafenib for the Treatment of Central Nervous System Metastases from BRAF V600 Mutated Metastatic Melanoma	Phase I	NCT05388877
Nivolumab With Trametinib and Dabrafenib, or Encorafenib and Binimetinib, in Treating Patients with BRAF Mutated Metastatic or Unresectable Stage III-IV Melanoma	Phase II	NCT02910700
A Study to Compare the Administration of Encorafenib + Binimetinib + Nivolumab Versus Ipilimumab + Nivolumab in BRAF-V600 Mutant Melanoma with Brain Metastases	Phase II	NCT04511013
Defactinib and Avutometinib, with or Without Encorafenib, for the Treatment of Patients with Brain Metastases from Cutaneous Melanoma	Phase I/II	NCT06194929
Immunotherapy		
Phase II Study of Nivolumab in Combination with Relatlimab in Patients with Active Melanoma Brain Metastases	Phase II	NCT05704647
A Study of LN-144 (TIL therapy) in People with Metastatic Melanoma to the Brain	Phase I	NCT05640193
Pembrolizumab Plus Bevacizumab for Treatment of Brain Metastases in Metastatic Melanoma or Non-small Cell Lung Cancer	Phase II	NCT02681549
Low Dose Ipilimumab with Pembrolizumab in Treating Patients with Melanoma That Has Spread to the Brain	Phase II	NCT03873818
Natural Killer Cell Therapy (UD TGFbetai NK Cells) and Temozolomide for the Treatment of Stage IV Melanoma Metastatic to the Brain	Phase I/II	NCT05588453
Study Comparing Investigational Drug HBI-8000 (Selective Histone Deacetylase Inhibitor) Combined with Nivolumab vs. Nivolumab in Patients with Advanced Melanoma	Phase III	NCT04674683
Bevacizumab and Atezolizumab With or Without Cobimetinib in Treating Patients with Untreated Melanoma Brain Metastases	Phase II	NCT03175432
Pembrolizumab and Lenvatinib in Patients with Brain Metastases from Melanoma or Renal Cell Carcinoma	Phase II	NCT04955743
Crizanlizumab Alone or in Combination with Nivolumab for Glioblastoma and Melanoma with Brain Metastases	Phase II	NCT05909618

## 5. Conclusions

A huge number of new targeted agents are now available in clinical trials in BM from NSCLC, breast cancer and melanoma, and increasingly the new molecular compounds display greater penetration into the brain. The post-hoc analyses of clinical trials on new drugs for established BM in order to define the risk of relapse into the brain after first-line treatment simply represent the initial step to investigate a preventive role, and they may serve to select the most promising compounds. Afterwards, well-designed prospective trials should follow to confirm the preliminary findings in terms of robustness.

When designing trials on the prevention of brain metastases, there are several critical issues to face. There is a need to identify subgroups of patients within each tumor type at a higher, preferably isolated, risk of CNS relapse. The agent to be tested for prevention should be able to cross the normal BBB and target micrometastases. In this regard, preclinical models for an early investigation of the potential efficacy of new compounds should be available before moving to clinical studies.

Moreover, window-of-opportunity trials (the so-called “phase zero trials”) should ideally precede phase II or III trials in order to better clarify the pharmacokinetic and pharmacodynamic properties of a new drug in tumor tissue obtained from resection following the investigational treatment.

For primary chemoprevention studies, high-risk patients with no history of CNS involvement should be selected. For secondary chemoprevention studies, one can select patients with a limited number of brain metastases after surgical resection or stable after radiosurgery to be treated with the investigational agent. In the future, new compounds able to target not only the micrometastases but also the early colonization of the brain will hopefully be increasingly available.

## Figures and Tables

**Table 3:** Ongoing trials on systemic treatment of brain metastases from NSCLC.

Study Name	Target	Phase	Trial
Targeted therapy			
Study of Osimertinib + SRS vs. Osimertinib Alone for Brain Metastases in EGFR-Positive Patients With NSCLC	EGFR	Phase II	NCT03769103
Keynatinib in Treated Patients With NSCLC and Brain Metastases	EGFR	Phase II	NCT04824079
A Randomised Phase II Trial of Osimertinib With or Without SRS for EGFR Mutated NSCLC With Brain Metastases (OUTRUN)	EGFR	Phase II	NCT03497767
A Phase I/II Study of AMG 510 in Combination With MVASI in Patients With Advanced, Unresectable or Metastatic KRAS G12C Mutant NSCLC With Asymptomatic Brain Metastasis	KRASG12C VEGF	Phase I Phase II	NCT05180422
Study of TY-9591 in Patients With a Lung Cancer With Brain or Leptomeningeal Metastases With EGFR Mutation	EGFR	Phase II	NCT05146219
Neurocognition in NSCLC Patients Treated With Osimertinib or Osimertinib + WBI	EGFR	Phase II	NCT04829019
Almonertinib Combined With Cerebral Radiation Treat Brain Metastases From EGFR-Positive NSCLC	EGFR	Phase II	NCT04905550
Immunotherapy			
Nivolumab and Ipilimumab Plus Chemotherapy for Patients With Stage IV Lung Cancer With Brain Metastases (NIVIPI-Brain)	CTLA-4 PD-1	Phase II	NCT05012254
Pembrolizumab Plus Bevacizumab for Treatment of Brain Metastases in Metastatic Melanoma or Non-small Cell Lung Cancer	PD-L1 VEGF	Phase II	NCT02681549
Phase II Investigation of Use of CNS Active Pembrolizumab and Chemotherapy for Asymptomatic Brain Metastasis From Non-small Cell Lung Cancer (NSCLC)	PD-L1	Phase II	NCT04964960
Toripalimab Combined With Anlotinib and SBRT in Patients With Untreated Brain Metastases of Driven Gene-negative NSCLC	Non-driver-related brain metastasis	Phase I	NCT05021328
LITT and Pembrolizumab in Recurrent Brain Metastasis (TORCH)	Recurrent brain metastasis with failed radiosurgery	Phase I	NCT04187872

**Table 6:** Ongoing trials on systemic treatment of brain metastases from HER2/HER3-positive breast cancer.

Study Name	Phase	NCT Number
HER2 targeted		
HER2-CAR T Cells in Treating Patients with Recurrent Brain or Leptomeningeal Metastases	Phase I	NCT03696030
Secondary Brain Metastases Prevention After Isolated Intracranial Progression on Trastuzumab/Pertuzumab or T-DM1 in Patients With advanced Human Epidermal Growth Factor Receptor 2+ Breast Cancer with the Addition of Tucatinib (BRIDGET)	Phase II	NCT05323955
Tucatinib, Trastuzumab and Capecitabine With SRS for Brain Metastases From HER-2-Positive Breast Cancer	Phase I	NCT05553522
Palbociclib, Trastuzumab, Pyrotinib and Fulvestrant Treatment in Patients with Brain Metastasis From ER/PR-Positive, HER-2-Positive Breast Cancer: A Multi-center, Prospective Study in China	Phase II	NCT04334330
Pyrotinib Combined with Capecitabine and Bevacizumab for Patients with HER2-Positive Breast Cancer and Brain Metastases	Phase II	NCT06152822
A Study of Pyrotinib Plus Capecitabine Combined with SRT in HER2+ MBC With Brain Metastases	Phase II	NCT05042791
Trial of Neratinib Plus Capecitabine in Subjects with HER2-Negative Metastatic Breast Cancer with Brain Metastases and Abnormally Active HER2 Signaling	Phase II	NCT04965064
GDC-0084 in Combination with Trastuzumab for Patients with HER2-Positive Breast Cancer Brain Metastases	Phase II	NCT03765983
Study of SHR-A1811 in HER2-expression Advanced Breast Cancer with Brain Metastases	Phase II	NCT05769010
A Study of Tucatinib Given Before Surgery to People with HER2+ Cancers That Have Spread to the Brain	Phase II	NCT05892068
Dendritic Cell Vaccines Against Her2/Her3 and Pembrolizumab for the Treatment of Brain Metastasis from Triple-Negative Breast Cancer or HER2+ Breast Cancer	Phase II	NCT04348747
HER3 targeted		
HER3-DXd in Breast Cancer and NSCLC Brain Metastases and Solid Tumor Leptomeningeal Disease (TUXEDO-3)	Phase II	NCT05865990

## Data Availability

No new data were created or analyzed in this study. Data sharing is not applicable to this review.
